# Superior Vena Cava Syndrome Due to Thymic Carcinoma

**DOI:** 10.7759/cureus.11670

**Published:** 2020-11-24

**Authors:** Jefferson Fabian Nieves Condoy, Luis Abraham Zúñiga Vázquez, Erick Martín Páez Hernández, Aldo Edyair Jiménez Herevia, Camilo Levi Acuña Pinzon

**Affiliations:** 1 Surgery, Hospital Regional de Alta Especialidad del Bajío, Leon, MEX; 2 Surgical Oncology, Hospital Regional de Alta Especialidad del Bajío, León, MEX; 3 Surgery, Hospital Regional de Alta Especialidad del Bajío, León, MEX; 4 General Surgery, Hospital Regional de Alta Especialidad del Bajío, León, MEX

**Keywords:** superior vena cava syndrome, thymic carcinoma, pancoast tumour

## Abstract

The superior vena cava syndrome (SVCS) is caused by a mechanical obstruction; 90% are of neoplasic etiology (lung cancer (LC) and non-Hodgkin lymphoma (NHL) mostly), epithelial neoplasms of the thymus (NET) is a rare cause, thymic carcinoma (TC) causing less than 1% of cases.

A 56-year-old male presented with a four-month history of dyspnea, dysphonia, facial and cervical edema and bilateral cervical lymphadenopathy. The tomography showed bilateral, mediastinal, retroperitoneal lymphadenopathies, and obstruction of the internal jugular vein, right apical pulmonary nodules. A superficial adenopathy biopsy was taken, which is not conclusive, so it was decided to take an image-guided biopsy. During its evolution, it presents asymptomatic cardiovascular changes; in extension studies, systemic disease is evidenced. The definitive histopathological study reported thymic carcinoma. Systemic treatment with chemotherapy and radiation therapy was planned.

## Introduction

The superior vena cava syndrome (SVCS) is caused by a mechanical obstruction; 90% are of neoplastic etiology (lung cancer (LC) and non-Hodgkin lymphoma (NHL) mostly), epithelial neoplasms of the thymus (NET) is a rare cause, and thymic carcinoma (TC) causes less than 1% of cases [1].

A 56-year-old male presented with a four-month history of dyspnea, dysphonia, facial and cervical edema, and bilateral cervical lymphadenopathy. The tomography showed bilateral, mediastinal, retroperitoneal lymphadenopathies, obstruction of the internal jugular vein, and right apical pulmonary nodules. A superficial adenopathy biopsy was taken, which was not conclusive, so it was decided to take an image-guided biopsy. During its evolution, it presented with asymptomatic cardiovascular changes; in extension studies, incluyed thorax, neck, and brain tomography; systemic disease also evidenced. The definitive histopathological study reported thymic carcinoma. Systemic treatment with chemotherapy and radiation therapy was planned.

NETS are currently considered systemic diseases that produce local and systemic symptoms [2], which broadens the differential diagnosis and makes an initial directed treatment difficult; they are frequently diagnosed in advanced stages which limits therapeutic options.

Thymic carcinoma is an uncommon etiology of SVCS, being associated with poor prognosis [3].

## Case presentation

**Figure 1:**
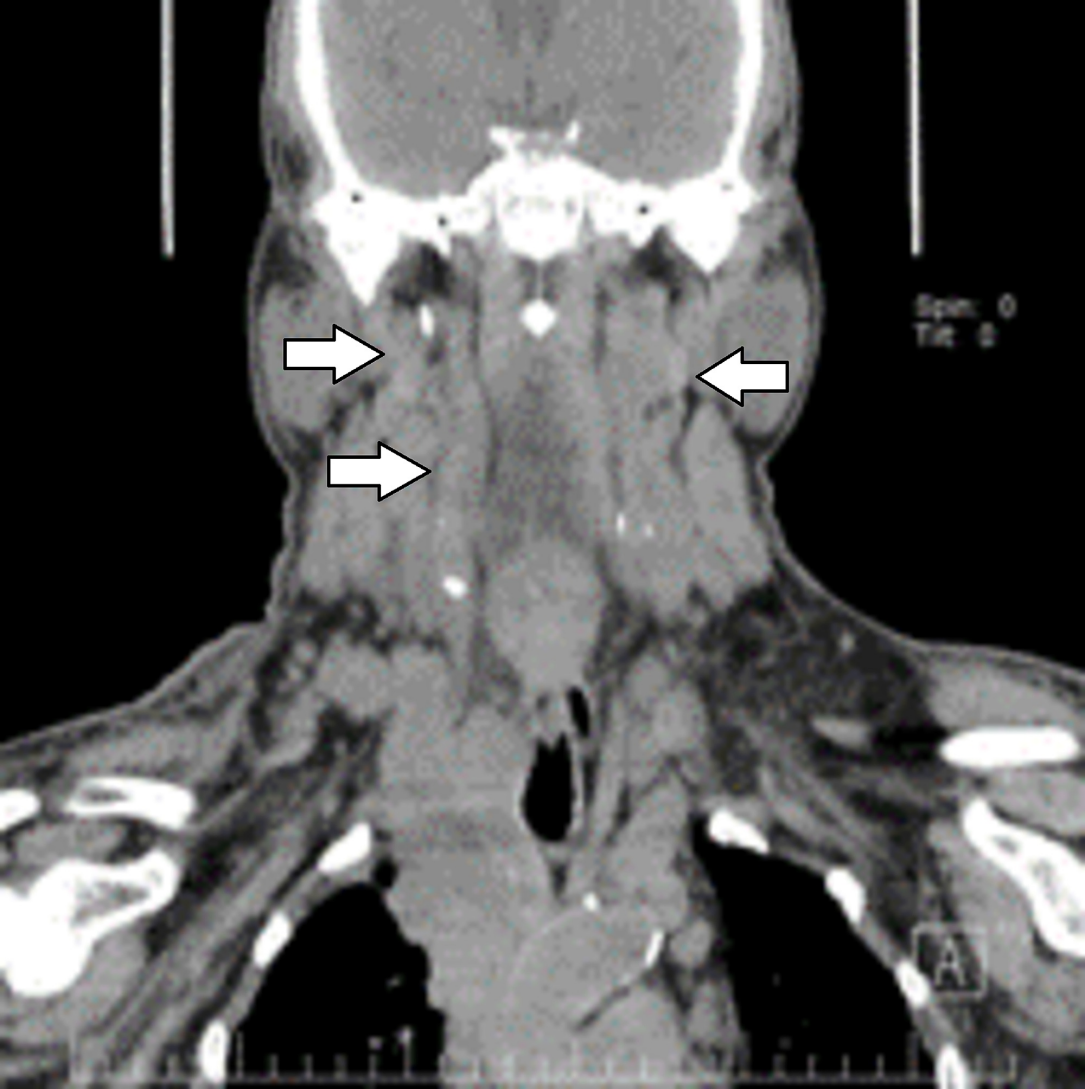
Coronal head and neck computed tomography. Bilateral adenopathy; in the right neck at levels IIA, IV (right arrows); left neck at level III (left arrow).

A 56-year-old male patient, with a family history of breast cancer, and a 40 pack-year smoker, four months ago presented with the clinical onset of dyspnea, dysphonia and progressive cranio-facial-thoracic-brachial edema that limited activities. He denied muscle weakness and associated muscular symptoms. Physical examination revealed head, neck, and thoracic limb edema and jugular engorgement with thoracic collateral circulation. Computed tomography revealed bilateral adenopathy predominantly in the right neck (levels IB, IIA, IV a and b) and in the right accessory spinal chain and left neck at level III (Figure 1); right internal jugular vein compressed by lymphadenopathies with filling defect in their lower third and dilatation of the left jugular vein.

**Figure 2:**
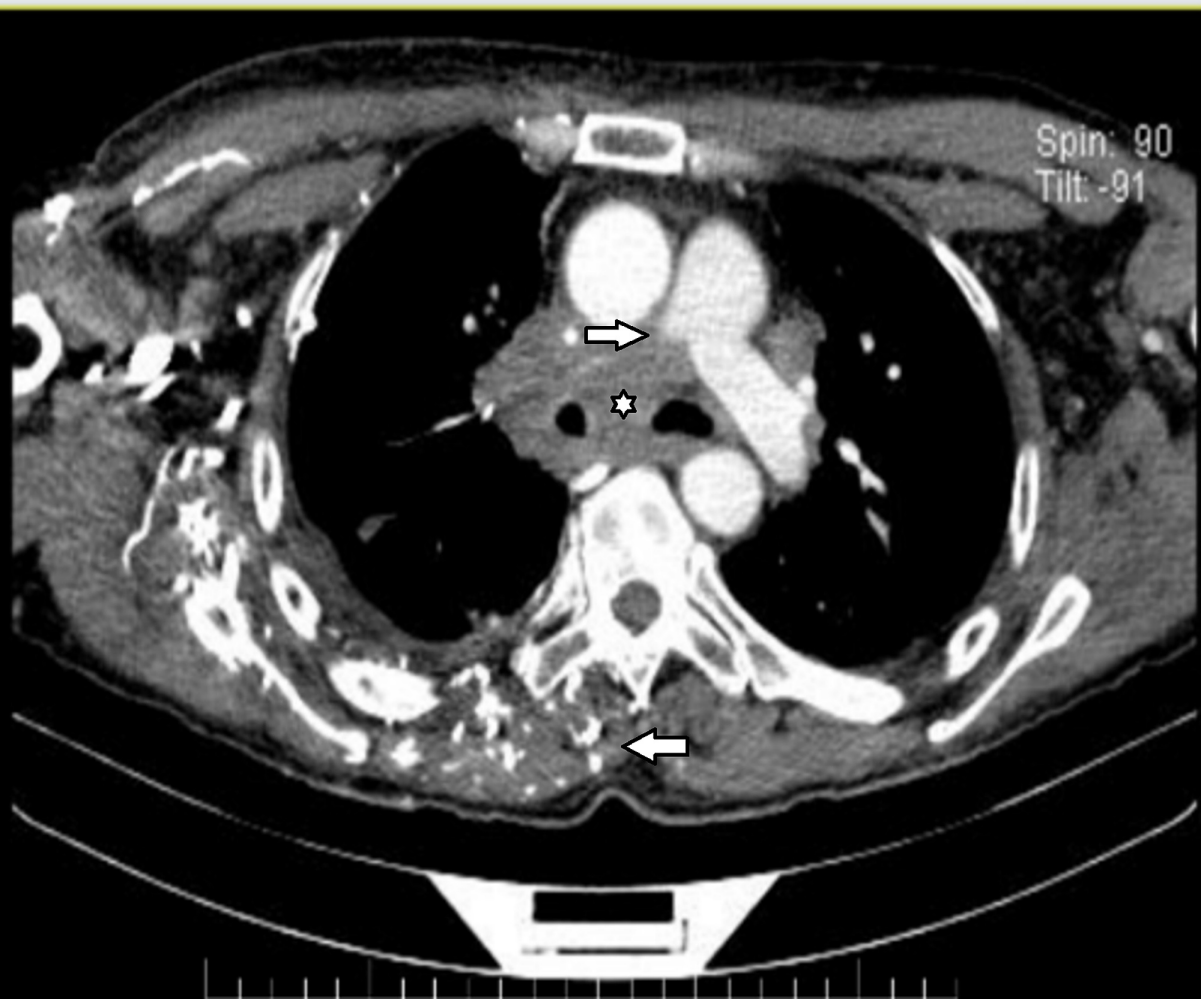
Mediastinum computed tomography. Arterial phase. Chest adenopathy in the right mediastinum that conditions displacement of the larynx and esophagus as well as compression of the brachiocephalic trunk (star) and right pulmonary artery with obstruction of more than 90% (top arrow) with collateral circulation (bottom arrow).

Chest adenopathy in the right mediastinum that conditions displacement of the larynx and esophagus as well as compression of the brachiocephalic trunk and right pulmonary artery with obstruction of more than 90% with collateral circulation (Figure 2).

**Figure 3:**
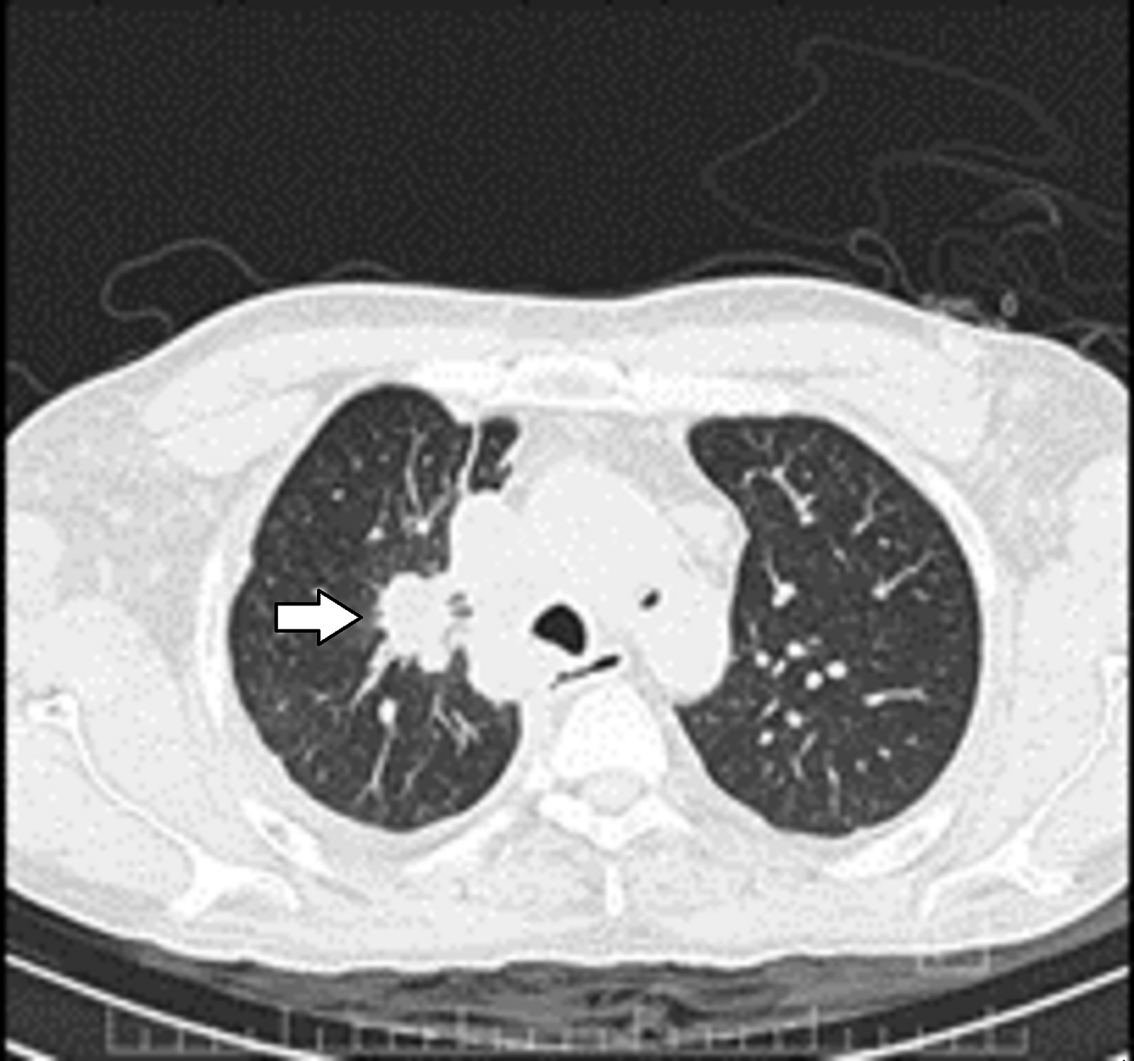
Axial computed tomography of the lung Parahiliar right lung mass in segment 1 with peripheral ground glass satellite lesions (arrow).

Parahiliar right lung mass in segment 1 with peripheral ground glass satellite lesions and right laminar pleural effusion (Figure 3); left retroperitoneal ganglionic conglomerate. He was admitted with a diagnosis of superior vena cava syndrome and probable Pancoast tumour.

Admission lab values: creatinine 0.5, urea 15, Na 135, K 4.0, Hb 13.8, leukocytes 10.9, platelets 325, carcinoembryonic antigen (CEA) 169, prostate specific antigen (PSA) 0.86, free PSA 0.14, thrombin 12.4 partial thromboplastin time 28.5. Rest of laboratory analysis with no alterations.

An adenopathy biopsy was performed with no complications. He received systemic corticosteroids and enoxaparin because of internal jugular thrombosis; the evidence of multiple lymphadenopathy in different regions raised the suspicion of a lymphoproliferative process, and the clinical response to systemic corticosteroids seemed to support this suspicion; however the pulmonary nodule raised diagnostic doubt. The initial biopsy result was inconclusive and an ultrasound-guided biopsy was carried out, and given the clinical improvement, he was discharged pending the biopsy result.

**Figure 4:**
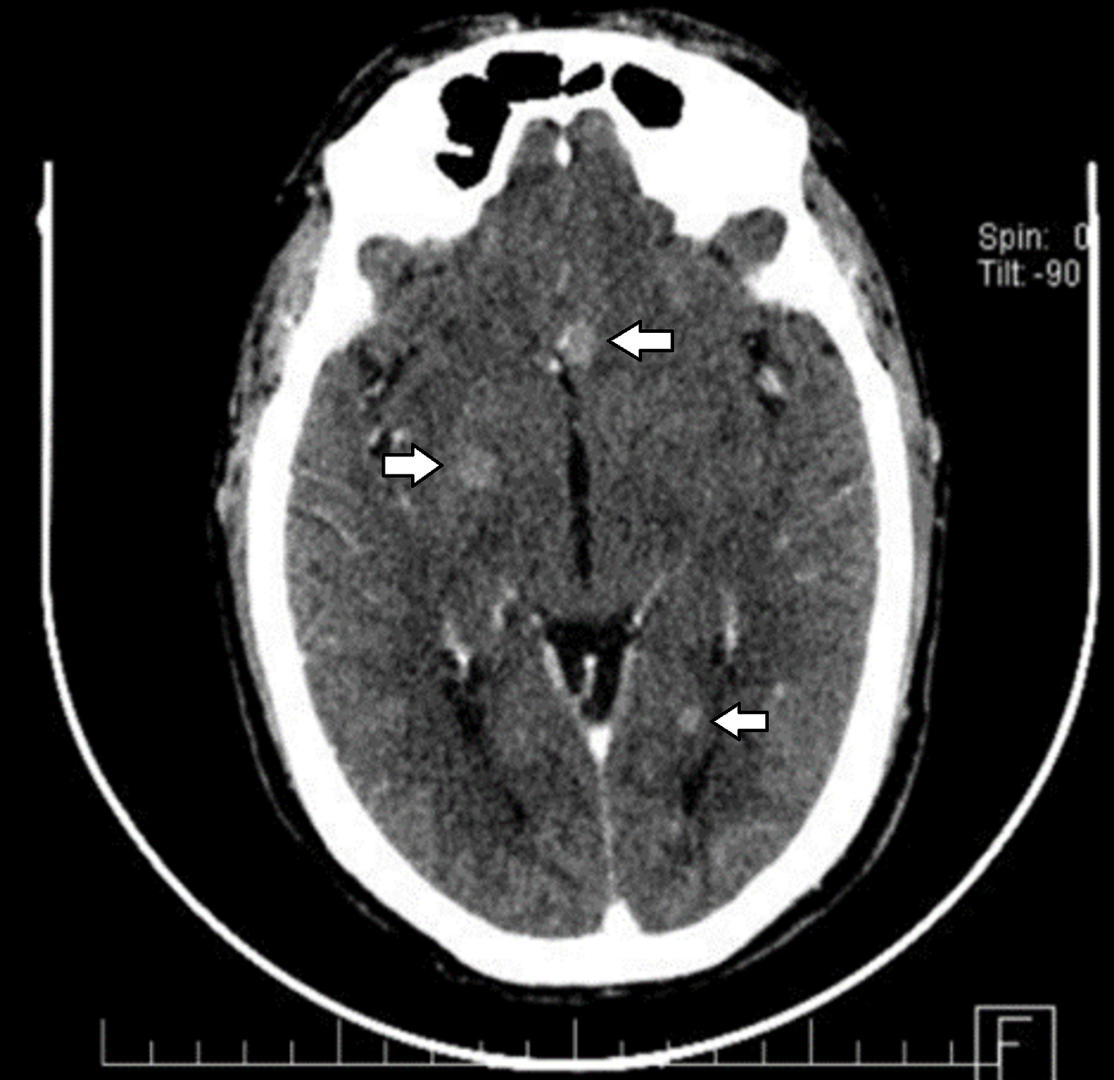
Computed tomography of the brain Three nodular brain lesions with contrast enhancement

He was readmitted one month later with neuropathic chest pain and edema in the chest, limitation of the range of motion of the neck that limited physical activity. Physical examination revealed the presence of rhonchi in the right hemithorax; imaging studies showed an increase in the volume of previously evidenced adenopathies. During his hospitalization, he developed arterial hypertension and atrial fibrillation, for which treatment with antihypertensives and class III antiarrhythmic drugs was initiated. A pericardial effusion of asymptomatic course developed. A diagnostic dilemma arose in view of the clinical picture that began with superior vena cava syndrome, evidenced by multiple lymphadenopathy in various regions, pulmonary nodule and initial response to systemic steroids; the preliminary pathology report did not show data of a lymphoproliferative process, which was corroborated with a negative CD 20; in the final pathology report it was identified as a thymic carcinoma. In the extension studies, nodular brain lesions with contrast enhancement were observed, they were asymptomatic (Figure 4). Treatment with radiotherapy was planned.

## Discussion

Superior vena cava syndrome (SVCS) was initially described by Hunter in 1757, in a patient with syphilitic aortic aneurysm; in the post-antibiotic era, the main etiology is neoplastic, as well as intravascular devices that are associated with thrombosis in cancer patients [1]. Thymic carcinoma is an uncommon etiology of SVCS, being associated with poor prognosis [3]. High alcohol consumption and smoking increase the risk of thymoma [4]. The pathophysiology of this syndrome is altered venous drainage of the skull, neck, thoracic limbs and upper thorax due to obstruction that will lead to the redirection of blood flow through multiple collateral veins towards the azygous, hemiazygous, intercostal, mediastinal, and paravertebral veins. Depending on the time of obstruction and time of diagnosis, patients with a gradual obstruction may be asymptomatic until the collateral network conforms properly. Conversely, if the obstruction is of rapid onset and almost complete then a different clinical picture may be present including dyspnea, swelling of the neck, trunk, face, chest pain, collateral venous distention, plethora and dysphagia. In SVCS head and neck edema will lead to a narrowing of the airways that can be life-threatening. Brain edema can occur which can lead to cerebral ischemia, herniation, and death. Cardiac output can be compromised leading to haemodynamic instability [5]. However, neoplasms of the thymus have been proposed as systemic diseases due to the coexistence of these various entities, as demonstrated by Weissferdt et al., when studying the clinical-pathological correlation of 1740 cases, of which 17% had a history of myasthenia gravis, 3.8% was associated with some autoimmune condition and 6.8% with another neoplasm, histologically 87.13% corresponded to thymomas and 12.7% were atypical thymomas; in post-resection follow-up, 10% had recurrence, 28.2% died from the tumor, and 71.8% died due to other causes [6,7].

Treatment in SVCS is conditioned by the presence of life-threatening conditions and comorbidities, the emergent treatment is aimed at minimizing acute cardiac and respiratory complications. There are scores to determine the urgency of SVCS: 5% correspond to the most serious life-threatening stage, the treatment of choice is stent placement that improves symptoms in 0-72 hours and thrombolytic therapy if indicated, with an 88% efficacy if started within the first five days after the thrombus has developed [2].

Although steroids and diuretics are frequently administered to reduce symptoms, their effectiveness has not been proven, so we have to take into account the side effects of these before indicating them. The use of endovascular stents is effective and fast in the recanalization of the vena cava regardless of its etiology, has an effectiveness that exceeds 90% at seven days; its the first line of treatment due to low complications [8].

Traditionally, radiotherapy is used as treatment in most cases with palliative intentions and shows improvement of symptoms in 80% of cases. Radiation can be used with high fractionation (300-400 cGy daily for three fractions) and conventional fractionation (200 cGy daily, five weekly fractions). Chemotherapy shows improvement of symptoms in 43% of patients treated initially treated with radiation and 73% of patients treated with induction chemotherapy [9].

## Conclusions

TC is a rare etiology of SVCS, in most cases it is a late stage associated with great morbidity and mortality. Its management depends on the clinical context at the time of diagnosis, with the stent being the first choice in cases of emergency, and radiotherapy and chemotherapy as planned treatment usually of a palliative nature.

## References

[1] Zimmerman S, Davis M (2018). Rapid fire: superior vena cava syndrome. Emerg Med Clin North Am.

[2] Straka C, Ying J, Kong F-M, Willey CD, Kaminski J, Kim DN (2016). Review of evolving etiologies, implications and treatment strategies for the superior vena cava syndrome. Springerplus.

[3] Salazar VR, Torrecillas LG, Contreras MH (2012). Superior vena cava syndrome as the initial manifestation of thymic carcinoma [Article in Spanish]. Arch Bronconeumol.

[4] Eriksson M, Kaerlev L, Johansen P (2019). Tobacco smoking and alcohol consumption as risk factors for thymoma - a European case-control study. Cancer Epidemiol.

[5] Pech-Alonso B, Fermín-Hernández C, Saavedra-de Rosas SI, Cicero-Sabido RJ (2018). Superior vena cava syndrome: clinical considerations [Article in Spanish]. Rev Med Hosp Gen (Mex).

[6] Weissferdt A, Kalhor N, Bishop JA (2018). Thymoma: a clinicopathological correlation of 1470 cases. Hum Pathol.

[7] Trujillo-Reyes JC, Martínez-Téllez E, Belda-Sanchis J (2019). Thymoma. A systemic disease? [Article in Spanish]. Arch Bronconeumol.

[8] Coiffard B, Elharrar X, Vandemoortele T, Laroumagne S, Dutau H, Astoul P (2014). The hypermetabolic mushroom: superior vena cava syndrome. Am J Med.

[9] Talapatra K, Panda S, Goyle S, Bhadra K, Mistry R (2016). Superior vena cava syndrome: a radiation oncologist’s perspective. J Cancer Res Ther.

